# Combination Usage of AdipoCount and Image-Pro Plus/ImageJ Software for Quantification of Adipocyte Sizes

**DOI:** 10.3389/fendo.2021.642000

**Published:** 2021-08-04

**Authors:** Yepeng Hu, Jian Yu, Xiangdi Cui, Zhe Zhang, Qianqian Li, Wenxiu Guo, Cheng Zhao, Xin Chen, Meiyao Meng, Yu Li, Mingwei Guo, Jin Qiu, Fei Shen, Dongmei Wang, Xinran Ma, Lingyan Xu, Feixia Shen, Xuejiang Gu

**Affiliations:** ^1^Department of Endocrine and Metabolic Diseases, The First Affiliated Hospital of Wenzhou Medical University, Wenzhou, China; ^2^Shanghai Key Laboratory of Regulatory Biology, Institute of Biomedical Sciences and School of Life Sciences, East China Normal University, Shanghai, China; ^3^Key Laboratory of Adolescent Health Assessment and Exercise Intervention of Ministry of Education, College of Physical Education and Health, East China Normal University, Shanghai, China

**Keywords:** adipocyte sizes, AdipoCount, Image-Pro Plus, ImageJ, obesity

## Abstract

In recent decades, the prevalence of obesity has been rising. One of the major characteristics of obesity is fat accumulation, including hyperplasia (increase in number) and hypertrophy (increase in size). After histological staining, it is critical to accurately measure the number and size of adipocytes for assessing the severity of obesity in a timely fashion. Manual measurement is accurate but time-consuming. Although commercially available adipocyte counting tools, including AdipoCount, Image-Pro Plus, and ImageJ were helpful, limitations still exist in accuracy and time consuming. In the present study, we introduced the protocol of combined usage of these tools and illustrated the process with histological staining slides from adipose tissues of lean and obese mice. We found that the adipocyte sizes quantified by the tool combination were comparable as manual measurement, whereas the combined methods were more efficient. Besides, the recognition effect of monochrome segmentation image is better than that of color segmentation image. Overall, we developed a combination method to measure adipocyte sizes accurately and efficiently, which may be helpful for experimental process in laboratory and also for clinic diagnosis.

## Introduction

Obesity results from energy intake in excess of energy expenditure, and surplus energy is stored in adipocytes as a form of triglycerides, which results in adipocyte hyperplasia and hypertrophy (1, 2). When the capacity of adipocytes cannot meet the demand for lipid storage, excess lipids will be ectopically deposited in the muscles, liver, and pancreas, causing metabolic abnormalities, such as insulin resistance (3, 4). Therefore, quantification of the number and size of adipocytes is essential for assessing the severity of obesity and providing the reference for the choice of treatment (5).

As technology advances, image processing software have been developed for the measurement of adipocytes (6–8). ImageJ and Image-Pro Plus (IPP) are both the open-software platform image processing tools for scientific image analysis and have been widely used in life sciences (9, 10). Area of interest (AOI) and measurement threshold of adipose tissue slides can be set to screen out objects that meet the requirements automatically (11, 12). However, because adipocytes are membrane-stained, and clear contrast or threshold is difficult to be set by these tools, the automatic counting of adipocyte sizes on adipose tissue slides may result in severe measurement errors. Meanwhile, drawing the adipocyte boundaries manually by IPP and ImageJ is accurate but time-consuming and intractable to large-scale investigations.

Researchers have been committed to developing software that can identify adipocytes accurately and efficiently (13, 14). However, these automatic methods may require programming knowledge or plug-ins, and the counting results also rely on slide quality, software recognition, so the accuracy is often unsatisfactory (15–18). Recently, a freely available counting software for adipocyte numbers, AdipoCount, is reported to be convenient and has powerful capability to recognize adipocyte boundaries and segment images with simple operations for further analysis (19). During our operation, we found that adipocytes with empty holes, cell debris, and cells adhesion edges may also be mistakenly counted by AdipoCount software. Besides, AdipoCount software lacks threshold settings and one-step area measurement function, thus the counting mistake could not be adjusted, and final results have to be calculated.

In the present study, we combined AdipoCount with IPP/ImageJ to measure the size of adipocytes while considering the following reasons: 1) IPP and ImageJ could not identify contours of adipocytes clearly but they could provide accurate results with clear segmentation and perform threshold setting to screen out unreasonable data; 2) AdipoCount could recognize adipocyte boundaries and export segmentation images, although without area measurement and threshold setting functions. Therefore, we took advantage of AdipoCount and IPP/ImageJ and designed the combination protocol for adipocyte sizes counting. By comparison of the combined method to manual method, we confirmed its accuracy and time saving characteristic, which suggested the advantage of applying AdipoCount and IPP/ImageJ combination for adipocyte size measurement.

## Materials and Methods

### Animal Studies

Mouse studies were performed according to the guidelines of the East China Normal University Animal Care and Use Committee. C57BL/6J male mice, purchased from Shanghai Research Center for Model Organisms, were housed at 23 ± 1°C on a 12-h light/12-h dark cycle with free access to food and water. 8-week-old male mice (n=5 per group) were fed with normal chow diet (NCD) (Research Diet, D10012G) or high fat diet (HFD) (Research Diet, D12492). All mice were sacrificed 3 months later and epididymal fat (eWAT) and inguinal fat (iWAT) tissues were dissected for histological analysis.

### Histology Processing and Image Capture

Adipose tissues were fixed in 10% buffered formalin for 12 h and dehydrated following a standard procedure. Then, the tissues were embedded in paraffin wax and sections (5-µm thick) were cut. Hematoxylin and eosin (H&E) staining was performed as previous report (17). Images were captured by a Nikon microscope instrument (MODEL ECLIPSE Ci-L) with a 20× objective using a Sony camera(DS-2000P, 20Mp 1” Color Sony Exmor CMOS SENSOR).

### Software

A 32/64-bit operating system (Windows7-10) was required for the established protocol. AdipoCount (version 1.0), a free open access software (http://www.csbio.sjtu.edu.cn/bioinf/AdipoCount/), was used to segment the membranes of adipocytes. Image-Pro Plus (version 6.0.0.260, Media Cybernetics Corporation, USA) and ImageJ (version 1.52a, NIH, USA) were used as image analysis software. These softwares (AdipoCount, ImageJ, IPP) have good compatibility in Windows7-10 system.

### Manual Measurement With IPP/ImageJ

#### IPP

##### Open Base Image

The image of interest was imported through “File → Open” option on the tool bar.

##### Set Scale

An image with a known linear scale bar was opened firstly. Image was calibrated by clicking on “Measure → Calibration → Spatial Calibration”. In the pop-up window, “µm” was selected as unit, and “image” was clicked to show the “position line”, which was dragged to make it completely coincide with the known linear scale bar. Subsequently, the known length of scale bar was input in the box to finish the calibration.

##### Measurement

The measurement scale was selected firstly through “Measure →Calibration → Select Spatial Calibration”. The adipocyte areas were depicted manually using “Measure → Count/Size”, then clicking “Edit → Draw/Merge Objects” in the tool bar of pop-up window. During the measurement, the image should be enlarged by 50%, which would make the segmentation results more accurate. In this process, the following objects were excluded: objects whose sizes were not in the range of 240 to 15,000 μm^2^, cells adhesion edges, empty holes without clear structure, and cell debris.

“View → Measurement Data” was selected to show the measurement results.

#### ImageJ

##### Open Base Image

The image of interest was imported through “File → Open” option in the tool bar.

##### Set Scale

An image with a known linear scale bar was opened firstly. The “Straight line” tool was chosen in the tool bar to draw a line, which was as long as the known linear scale bar. “Analyze → Set scale” option was clicked and the distance of known linear scale was displayed in the “Known distance” box of the “Set Scale” window. The pixel aspect ratio was set to 1. The known distance of the linear scale bar was entered into the “Known Distance” box. The unit was dependent on the scale bar (such as µm). The “Global” option was selected to maintain the calibration for all subsequent image analysis if all images were taken at the same magnification.

##### Subtract Background

To help clarify the image for subsequent analysis, the backgrounds of images were corrected. “Process → Subtract Background” was selected from the task bar. “Rolling Ball Radius” should be set to 50 pixels. “Light Background” and “Sliding Paraboloid” should be checked. When all of the parameters were set, “OK” was clicked.

##### Measurement

The image should also be enlarged by 50%. The “Wand” (tracing tool) in the tool bar was chosen to select the individual adipocytes. However, this tool could not identify adipocytes accurately sometimes. For example, partial membranes of adipocytes were lost. Then, the “Polygon Selection” tool was select from the task bar to depict the adipocyte areas. Every time when a cell was depicted, “M” was pressed in keyboard, and the selected area would be measured. To avoid repeated measurements, “Backspace” was pressed to mark the cells which had been counted. Above operations were repeated until the last cell. The exclusion criteria were the same as IPP measurement.

### General Measurement (Identifying Adipocytes by IPP/ImageJ Directly)

#### IPP

##### Open Base Image and Set Scale

Same as described in “Manual measurement with IPP/ImageJ (2.4.1.)”.

##### Threshold Images

“Count/Size” option was selected under “File” drop-down menu. In the “Count/Size” window, the “Manual → Select Ranges” was clicked to show segmentation menu. “Histogram Based” option was selected in segmentation menu. The “HSI” was selected. In “H” and “S” channels, the first and second sliding bars were set to 0 and 255 respectively. As for “I” channel, the first sliding bar was set to 0, the second sliding bar was set to make the red highlight maximally cover the areas defined as adipocytes without impact on the membrane areas.

##### Measurement

The threshold of area was set through “Measure → Select measurements” in the “Count/Size” window. The filter range of area was set starting from 240 µm^2^ and ending at 15000 µm^2^. “Apply Filter Ranges” option was selected to apply the settings. Clicking “Count”, the software would measure the areas of objects.

#### ImageJ

##### Open Base Image, Set Scale, and Subtract Background

Same as described in “Manual measurement with IPP/ImageJ (2.4.2)”.

##### Threshold Images

The images were converted into 8-bit using “Image → Type → 8-bit” option. Then the “Image → Adjust → Threshold” was selected from the tool bar. The first sliding bar was set to 0, the second sliding bar was set to make the red highlight maximally covering the areas defined as adipocytes without impact on the membrane areas.

##### Measurement

The areas were measured through “Analyze → Analyze Particles”. In the pop-up window, the threshold of area, 240 to 15,000 µm^2^, was input into “Size” box. The options of “Display results”, “Exclude on edges” were selected. Clicking “OK”, the software would detect the areas.

### Adipocyte Boundary Segmentation by AdipoCount

The adipocytes were segmented by AdipoCount as previously described (19). Briefly, the image was imported through “File → Input image”. The “Re-segment” option was selected to improve accuracy, and then “Process” button was clicked to get preliminary segmentation image (**Supplementary Figure 1B**). The segmentation results could be further corrected manually. Missing membrane segments were added and false membrane segments were deleted. Moreover, holes with large areas should be separated into several small holes by “Add line”, then these small holes could be excluded in further detection through area threshold. Finally, two different segmentation images (monochrome and color) were exported by clicking “Counting → Save” in “Counting” box (**Supplementary Figures 1C, D**).

### Combined Methods for Measurement

#### Combination Usage of AdipoCount and IPP

##### Open Images and Select Calibrated Scale Bar

The monochrome and color segmentation images exported by AdipoCount were imported into IPP and the measure scale was selected as described above.

##### Threshold Images

“Count/Size” option was selected under “File” drop-down menu. In the “Count/Size” window, the “Manual → Select Ranges” was clicked to show segmentation menu.

The next steps were slightly different about monochrome and color segmentation images. For monochrome segmentation images, the first sliding bar was set to 52 (this varied until the red highlight covered all the empty space defined as adipocytes), the second sliding bar was set to 255. For color segmentation images, “Histogram Based” option was selected in segmentation menu. The “HSI” was selected. In “H” and “S” channels, the first and second sliding bars were set to 0 and 255, respectively. As for “I” channel, the first sliding bar was set to 0, the second sliding bar was set to make the red highlight maximally covering the areas defined as adipocytes without impact on the membrane areas.

##### Measurement

“Rectangular AOI” tool in task bar was selected to choose the area, which only contained intact cells. Then the “Options” was clicked, and “All Borders” was selected in “Clean Borders” box. In this way, the cells touching edge of the field would be excluded in next detections.

The threshold setting and the following operations were same as described in “General measurement (Identifying adipocytes by IPP/ImageJ directly) (2.5.1)”.

#### Combination Usage of AdipoCount and ImageJ

Before measurement, the quality of the slices needs to be manually checked: the cell boundaries in the slices are clear and there are enough cells for the measurement. In this study, we ensured that the number of cells in each field was not less than 25.

##### Open Images and Set Scale

The monochrome and color segmentation images were imported into ImageJ and the measure scale was set as described above.

##### Threshold Images

The images were converted into 8-bit using “Image → Type → 8-bit” option. Then the “Image → Adjust → Threshold” was selected from the tool bar.

The next steps were slightly different about monochrome and color segmentation images. For monochrome segmentation images, the first sliding bar was set to 52 (this varied until the red highlight covered all the empty space defined as adipocytes), the second sliding bar was set to 255. For color segmentation images, the first sliding bar was set to 0, the second sliding bar was set to 240 (this varied to make the red highlight maximally covering the areas defined as adipocytes without impact on the membrane areas).

After these steps, membranes and adipocytes areas were filled by white and black respectively.

##### Measurement

To exclude the cells touching edge of the field, “Rectangle” tool in task bar was used to select the area, which only contained intact cells (the area of rectangle would change to contain all intact cells). The areas were measured through “Analyze → Analyze Particles”. In the pop-up window, the threshold of area, 240-15000 µm^2^, was input into “Size” box. The options of “Display results” and “Exclude on edges” were selected. Clicking “OK”, the software would detect the areas.

The results would be presented in “Results” window.

Note: The Steps (2–3) could be automated by creating a macro. “Plugins → New → Macro” was selected and the computer script as following was pasted into a new screen. The new macro was saved by clicking “File → Save”. Then the subsequent image analysis could be performed easily using the created macro by clicking “Plugins → Macros → Run”.

The macro was different for monochrome and color segmentation images.

For monochrome segmentation images:

**run(“8-bit”);**
**setAutoThreshold(“Default”);**
**//run(“Threshold…”);**
**setThreshold(52, 255);**
**run(“Convert to Mask”);**
**//setTool(“rectangle”);**
**makeRectangle(48, 32, 5336, 3552);**
**run(“Analyze Particles…,” “size=240-15000 show=[Bare Outlines] display exclude summarize add in_situ”);**
 For color segmentation images:
**run(“8-bit”);**
**setAutoThreshold(“Default”);**
**//run(“Threshold…”);**
**setThreshold(0, 240);**
**setOption(“BlackBackground,” false);**
**run(“Convert to Mask”);**
**//setTool(“rectangle”);**
**makeRectangle(48, 40, 5352, 3576);**
**run(“Analyze Particles…,” “size=240-15000 show=[Bare Outlines] display exclude summarize add in_situ”);**

Note: The area of “Rectangle” should be adjusted and recorded when size of image changes. Then this new area should be used in macro to replace the old one. By this way, the macro could work when the size of images changed.

Videos were made to describe the details of the combined methods (**Supplementary Video**).

### Statistical Analysis

The number and size of adipocytes were measured by IPP or ImageJ. 2 slides were collected for each mouse, and 4 different fields were counted for each slide. Any objects with an area below 240 μm^2^ or above 15000 μm^2^ were excluded. In this study, the number of adipocytes for different tissues counted by each method was shown in **Supplementary Tables 1****–****4**. The frequency distribution of size was calculated from 240 to 15000 μm^2^ (this range might change for different animal model and tissue) in 500 increments. The number of adipocytes within the distribution was counted and the result was transformed to a percentage of total adipocytes in each group. A Student *t* test was performed to compare between two groups. Two-way ANOVA followed by a Bonferroni *post hoc* analysis was used for multiple comparisons in Prism 8 (GraphPad Software Inc.).

## Results

### General Measurement Results of IPP and ImageJ Are Not Accurate

**Figure 1:**
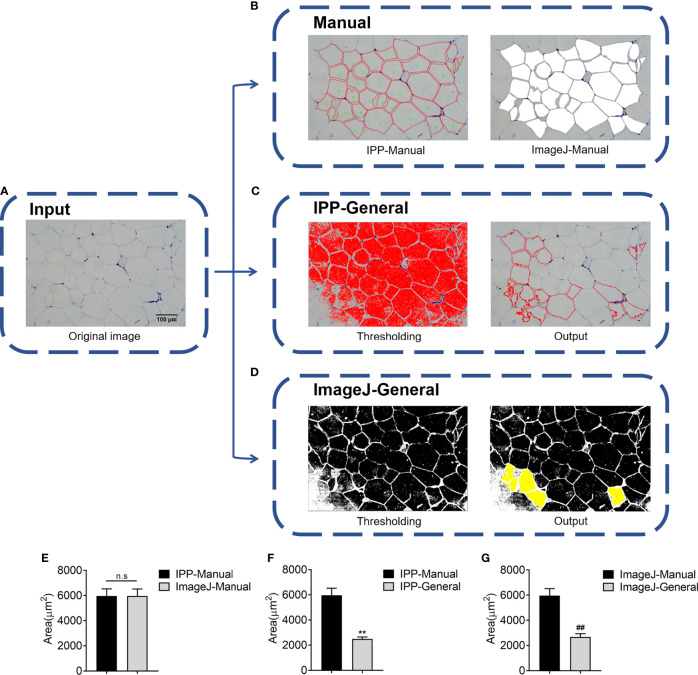
Adipocyte sizes measured by manual and general methods respectively with IPP/ImageJ. **(A)** Original image of adipocytes. **(B)** Manual segmentation results of IPP (left) and ImageJ (right). **(C)** General segmentation results of IPP, the image after threshold adjustment (left), and the output image, where the red circles represented identified adipocytes (right). **(D)** General segmentation results of ImageJ, the image after threshold adjustment (left), and the output image, where the identified adipocytes were filled with yellow (right). **(E)** Adipocyte sizes measured by manual methods with IPP and ImageJ. **(F)** Adipocyte sizes measured by manual and general methods with IPP. **(G)** Adipocyte sizes measured by manual and general methods with ImageJ. Data are presented as mean ± SEM and **P < 0.01 compared to IPP manual measurement group. ***^##^****P* < 0.01 compared to ImageJ manual measurement group. n.s., not significant; IPP, Image-Pro Plus.

In this study, two eWAT histological slides were selected to evaluate whether IPP or ImageJ could detect the area of adipocytes accurately using general methods. H&E staining of eWAT was measured by manual and general methods respectively with IPP/ImageJ. As shown in **Figures 1A–D**, IPP and ImageJ could not identify and segment adipocytes accurately by themselves, compared with manual methods. In detail, there was no significant difference between manual measurement results of IPP and ImageJ (**Figure 1E**), whereas the area measured by general methods was significantly smaller than the manual measurement result (**Figures 1F, G**). These results indicated that the general methods of IPP and ImageJ were unable to measure the area of adipocytes accurately.

### AdipoCount Cannot Recognize Empty Holes, Cell Debris, and Adhesion Edges

**Figure 2:**
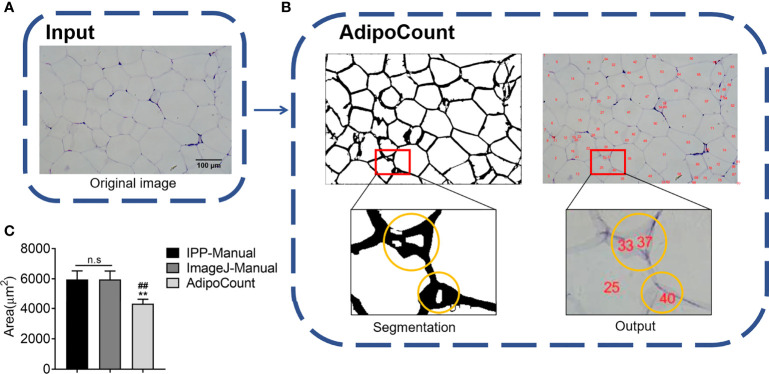
AdipoCount cannot recognize empty holes, cell debris, and adhesion edges. **(A)** Original image of adipocytes. **(B)** The segmentation image of AdipoCount (left), and the labeled image with cell numbers (right); what in the yellow circles were cell debris or empty holes (left), which were counted as cells mistakenly by AdipoCount (right). **(C)** Adipocyte sizes measured by manual methods with IPP and ImageJ, or calculated with AdipoCount. Data are presented as mean ± SEM and **P < 0.01 compared to IPP manual measurement group. ***^##^****P* < 0.01 compared to ImageJ manual measurement group. n.s., not significant; IPP, Image-Pro Plus.

AdipoCount is a software designed for adipocyte counting, which is powerful to segment adipocyte and export the image, although it only gives numbers but not areas directly. Thus, we next examined whether dividing the total area of the eWAT image previously tested by the number of cells could get an accurate area. An original image was imported into AdipoCount (**Figure 2A**), and the segmentation image revealed that empty holes, cell debris, and adipocyte adhesion edges were counted mistakenly (**Figure 2B**). Because the software could not set the threshold and manual calibration could not eliminate the errors, the counting number result was significantly more than the actual number. So, the adipocyte areas achieved was significantly underestimated (**Figure 2C**). These results suggested that there was still improvement space for AdipoCount to better calculate the adipocyte area.

### Combination of AdipoCount and IPP/ImageJ Identifies and Segments Adipocytes Accurately

*Via* the above tests, we found that IPP/ImageJ could set the threshold and had the area measurement function, although the ability to recognize adipocyte boundaries was poor during general measurement. On the other hand, AdipoCount had no measurement functions and threshold settings, although it could segment adipocytes with a high accuracy. Indeed, a preliminary segmentation image was generated by AdipoCount, which could be further corrected manually (**Supplementary Figure 1B**). Besides, from two different segmentation images (monochrome and color segmentation image) exported from AdipoCount, we could clearly see that adipocytes had clear boundaries in both two segmentation images (**Supplementary Figures 1C, D**). In addition, the size of segmentation images exported by AdipoCount was same as the original image. Images analyzed by the different software remained their original size of 5440 × 3648 pixels, suggesting that AdipoCount processing did not compress the image, and the calibration scale of original image could be used for segmentation image.

**Figure 3:**
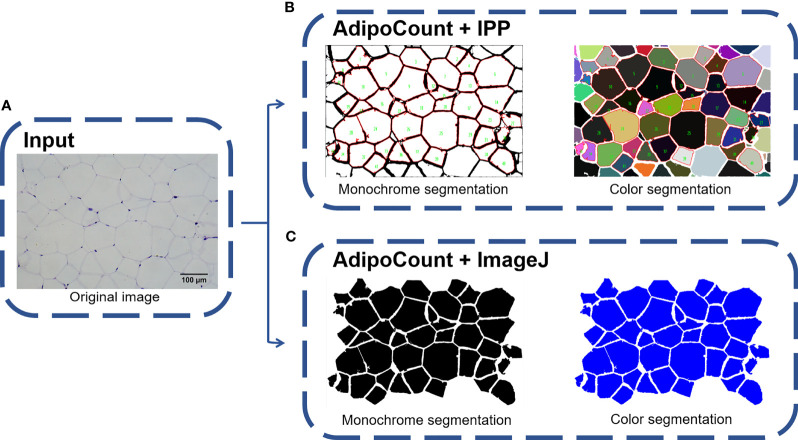
Combination of AdipoCount and IPP/Image identifies adipose cells accurately. **(A)** Original image of adipocytes. **(B)** Combination of AdipoCount and IPP, the identification results of monochrome segmentation image (left), and color segmentation image (right). **(C)** Combination of AdipoCount and ImageJ, the identification results of monochrome segmentation image (left), and color segmentation image (right). IPP, Image-Pro Plus.

We next imported both monochrome and color segmentation images into IPP and ImageJ to measure the adipocyte size. Of note, adipocytes in segmentation images could be identified accurately using general methods of IPP/ImageJ. Moreover, empty holes, cell debris, and adhesion edges were eliminated by setting threshold to area of interest (AOI) (**Figures 3A–C**). These results suggested that combination of AdipoCount and IPP/ImageJ might be a better method to measure the adipocyte sizes accurately.

### The Combined Methods Can Measure the Area Accurately and Take Less Time

**Figure 4:**
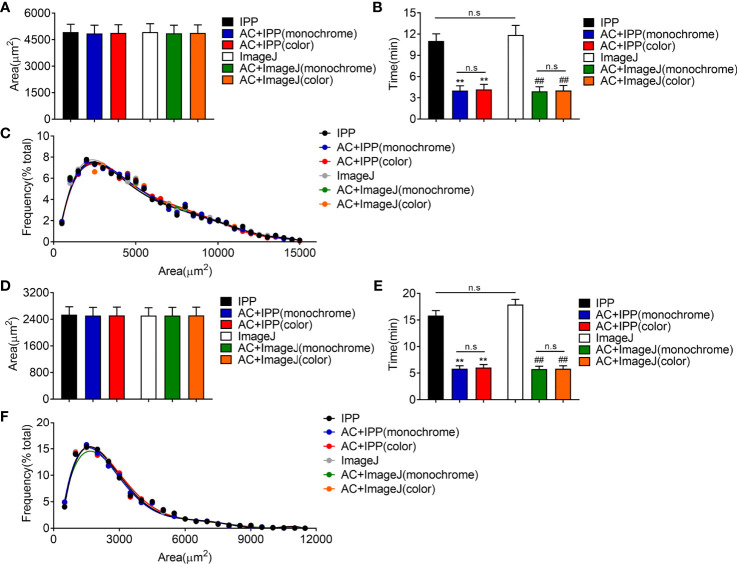
The combined methods can measure the area accurately and take less time in eWAT and iWAT of obese mice. **(A–C)** The measurement results of eWAT, adipocyte sizes derived from manual and combined methods **(A)**, measurement time **(B)**, adipocyte size distributions measured by manual and combined methods **(C)**. **(D–F)** The measurement results of iWAT. Data are presented as mean ± SEM and **P < 0.01 compared to IPP manual measurement group. ***^##^****P* < 0.01 compared to ImageJ manual measurement group. n.s., not significant; IPP, Image-Pro Plus; AC, AdipoCount.

Next, manual methods and combined methods were performed to examine the adipocyte sizes in eWAT and iWAT of obese mice. The average adipocyte sizes and size distributions in eWAT measured by combined methods were comparable with the manual measurement results (**Figures 4A, C****)**. Besides, there were no obvious differences between the measurement results of monochrome and color segmentation images (**Figures 4A, C****)**. Of note, the combined methods could significantly save measurement time (**Figure 4B**). In addition, the measurement results in iWAT were similar as those in eWAT (**Figures 4D–F**).

**Figure 5:**
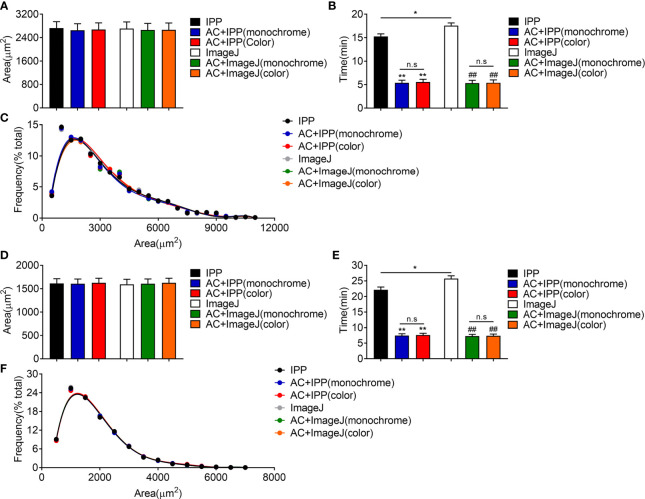
The combined methods can measure the area accurately and take less time in eWAT and iWAT of lean mice. **(A–C)** The measurement results of eWAT, adipocyte sizes derived from manual and combined methods **(A)**, measurement time **(B)**, adipocyte size distributions measured by manual and combined methods **(C)**. **(D–F)** The measurement results of iWAT. Data are presented as mean ± SEM and *P < 0.05, **P < 0.01 compared to IPP manual measurement group. ***^##^****P* < 0.01 compared to ImageJ manual measurement group. n.s, no significant. IPP, Image-Pro Plus; AC, AdipoCount.

Then, the combined methods were used to measure adipocyte sizes in eWAT and iWAT of lean mice. Similar to the results in obese mice, the combined methods could count the areas and size distributions accurately in lean mice, but saved time significantly (**Figures 5A–F**). Interestingly, we noticed that the manual measurement of ImageJ took more time than IPP, which was because of the different operating procedures of these two softwares (**Figures 5B, E**).

Very small adipocytes may lead to increased incidence of mis-counting, thus we further analyzed the size distributions in range of 0 to 1000 µm^2^. The results showed that the size distributions measured by the combined methods were comparable as the manual methods (**Supplementary Figures 2A–D**).

### Limitation and Suggestion of the Combined Measurement

**Figure 6:**
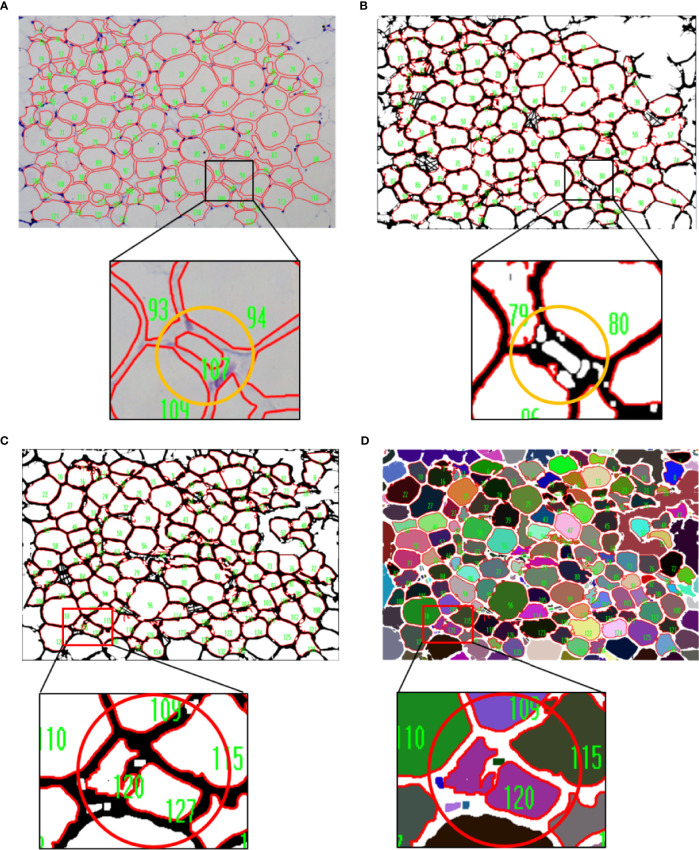
Limitation of the combined measurement. **(A, B)** The results of manual and combined measurement. The cell numbered 107 in yellow circle was counted when using manual measurement **(A)**,whereas it was excluded when the combined method was performed **(B)**. **(C, D)** Comparison of measurement results of two different segmentation images. Two cells inside the red circle could be distinguished in monochrome segmentation image **(C)**, but not in color segmentation image **(D)**.

During the operation, we noticed that there were small adipocytes with the size close to the lower limit of threshold exclusion in iWAT of lean mice. The result of UCP-1 immunostaining indicated the small adipocytes might be caused by the process called “browning of white fat”, which was important for lipid mobilization and thermogenesis (**Supplementary Figure 3**). These adipocytes were counted when using the manual measurement of IPP but excluded while the combined method was performed (**Figures 6A, B**), which might be due to the slight loss of area during segmentation by AdipoCount and the setting of the intensity thresholding. Of note, the combined measurement results were comparable to manual measurements, suggesting that individual cell misidentification had little effect on the overall result.

In a color segmentation image, if boundary between two adjacent adipocytes was thin and was filled with similar color, then the two adipocytes might be identified as one adipocyte in subsequent measurements. Although the area could be split in two manually, it would take more time. Thus, the monochrome segmentation image was better recognized and recommended (**Figures 6C, D**).

### Adipocyte Counting by the Combined Methods Yields Comparable Data as Manual Methods But Is More Efficient

**Figure 7:**
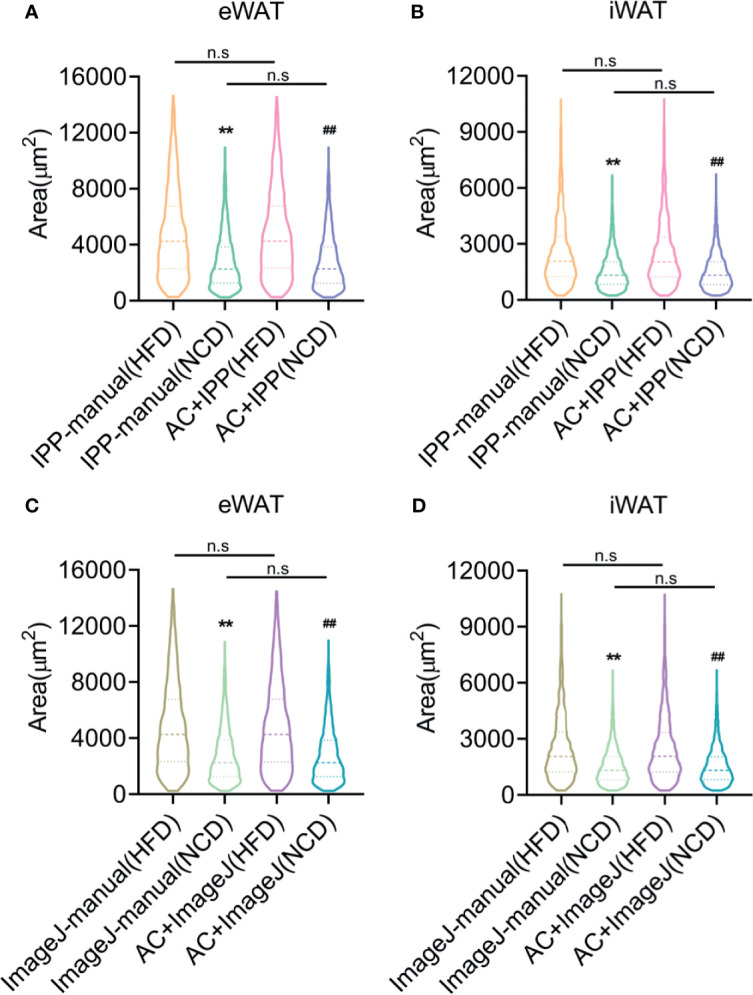
Adipocyte counting by the combined methods yields comparable data as manual methods but saves more time. **(A, B)** Adipocyte sizes measured by manual methods with IPP and combined methods with IPP and AdipoCount, in eWAT **(A)** and iWAT **(B)**. **(C, D)** Adipocyte sizes measured by manual methods with ImageJ and combined methods with ImageJ and AdipoCount, in eWAT **(C)** and iWAT **(D)**. Data are presented as mean ± SEM and **P < 0.01 compared to manual methods group. ***^##^****P* < 0.01 compared to combined methods group. n.s, no significant. IPP, Image-Pro Plus; AC, AdipoCount.

Finally, the combined methods using monochrome segmentation images were performed to measure the adipocyte sized in lean and obese mice. Both manual and combined methods identified a significant increase of adipocyte sizes and frequency of large adipocyte in fat tissues isolated from obese mice compared with lean mice (**Figures 7A–D**), with comparable area counting results, suggesting that the combined methods yielded the accurate results as manual methods with more efficiency.

## Conclusion

The measurement of adipocyte sizes is important for obesity research. In the present study, we provided an easy and semiautomated method by taking advantage of AdipoCount and IPP/ImageJ software to accurately and efficiently quantify adipocyte sizes. Briefly, adipocytes from H&E staining slides are segmented by AdipoCount, and clear segmentation images are exported. IPP or ImageJ is then performed to analyze segmentation images with threshold setting and one-step quantification. It has to be noticed that the monochrome image is more accurate than colored one. For the simple operation protocol, no programming experience is required. However, this method is not yet fully automated and still requires some manual manipulation, especially the manual correction of segmentation images from AdipoCount. The quality of slides, such as clear cell staining and complete membrane, is also important to get the accurate results, especially slides with small adipocytes. Further adjustment of the exclusion range and intensity threshold is required to improve the accuracy of the measurement. Theoretically, the combined method can be used for images with clear cell boundaries. More efforts will be made to explore whether this method can be applied besides adipocytes in the future.

## Data Availability Statement

The original contributions presented in the study are included in the article/**Supplementary Material**. Further inquiries can be directed to the corresponding authors.

## Ethics Statement

The animal study was reviewed and approved by the East China Normal University Animal Care and Use Committee.

## Author Contributions

XG, FeixiaS and LX conceived the project and designed the experiments. YH and JY carried out most of the experiments. XCu, ZZ, QL, and WG assisted in histology processing and image capture. CZ, XCh, MM, and YL provided rodent biological samples for association analysis. MG, JQ, FeiS, DW, and XM assisted in software manipulation and technical support. XG, FeixiaS and LX wrote and edited the paper. All authors contributed to the article and approved the submitted version.

## Funding

This project is supported by fundings from Key Research and Development Project of Zhejiang Province (2021C03069), Natural Science Foundation of Zhejiang Province (LY20H070003), National Key Research and Development Program of China (2019YFA0904500), National Natural Science Foundation of China (31800989, 32071148, 81902980), and ECNU public platform for Innovation (011), the Instruments Sharing Platform of School of Life Sciences, East China Normal University.

## Conflict of Interest

The authors declare that the research was conducted in the absence of any commercial or financial relationships that could be construed as a potential conflict of interest.

## Publisher’s Note

All claims expressed in this article are solely those of the authors and do not necessarily represent those of their affiliated organizations, or those of the publisher, the editors and the reviewers. Any product that may be evaluated in this article, or claim that may be made by its manufacturer, is not guaranteed or endorsed by the publisher.

## Supplementary Material

Supplementary Figure 1 Two different segmentation images of AdipoCount. **(A)** Original image of adipocytes. **(B)** Preliminary segmentation image without correction. **(C)** Monochrome segmentation image after correction. **(D)** Color segmentation image after correction.

Supplementary Figure 2 The combined methods measured the size of very small adipocyte accurately. **(A-D)** The size distributions of adipocytes (range of 240-1000µm2, in 100 increments) measured by manual and combined methods, in eWAT of obese mice **(A)**, in iWAT of obese mice **(B)**, in eWAT of lean mice **(C)**, in iWAT of lean mice **(D)** IPP, Image-Pro Plus; AC, AdipoCount.

Supplementary Figure 3 UCP-1 immunostaining of iWAT in lean mice.

## References

[1] Ghaben AL Scherer PE. Adipogenesis and Metabolic Health. Nat Rev Mol Cell Biol (2019) 20:242–58. 10.1038/s41580-018-0093-z 30610207

[2] Haczeyni F Bell-Anderson KS Farrell GC. Causes and Mechanisms of Adipocyte Enlargement and Adipose Expansion. Obes Rev (2018) 19:406–20. 10.1111/obr.12646 29243339

[3] Girousse A Virtue S Hart D Vidal-Puig A Murgatroyd PR Mouisel E. Surplus Fat Rapidly Increases Fat Oxidation and Insulin Resistance in Lipodystrophic Mice. Mol Metab (2018) 13:24–9. 10.1016/j.molmet.2018.05.006 PMC6026316 29789270

[4] Karpe F Dickmann JR Frayn KN. Fatty Acids, Obesity, and Insulin Resistance: Time for a Reevaluation. Diabetes (2011) 60:2441–9. 10.2337/db11-0425 PMC3178283 21948998

[5] Muir LA Neeley CK Meyer KA Baker NA Brosius AM Washabaugh AR. Adipose Tissue Fibrosis, Hypertrophy, and Hyperplasia: Correlations With Diabetes in Human Obesity. Obes (Silver Spring) (2016) 24:597–605. 10.1002/oby.21377 PMC4920141 26916240

[6] Chen HC Farese RV Jr. Determination of Adipocyte Size by Computer Image Analysis. J Lipid Res (2002) 43:986–9. 10.1016/S0022-2275(20)30474-0 12032175

[7] Maguire AS Woodie LN Judd RL Martin DR Greene MW Graff EC. Whole-Slide Image Analysis Outperforms Micrograph Acquisition for Adipocyte Size Quantification. Adipocyte (2020) 9:567–75. 10.1080/21623945.2020.1823139 PMC7714435 32954932

[8] Wiggenhauser PS Kuhlmann C Blum J Giunta RE Schenck T. Influence of Software Parameters on Measurements in Automatized Image-Based Analysis of Fat Tissue Histology. Acta Histochem (2020) 122:151537. 10.1016/j.acthis.2020.151537 32197756

[9] Schindelin J Rueden CT Hiner MC Eliceiri KW. The ImageJ Ecosystem: An Open Platform for Biomedical Image Analysis. Mol Reprod Dev (2015) 82:518–29. 10.1002/mrd.22489 PMC5428984 26153368

[10] Mei Y Tang Z Li Z Yang X. Repeatability and Reproducibility of Quantitative Corneal Shape Analysis After Orthokeratology Treatment Using Image-Pro Plus Software. J Ophthalmol (2016) 2016:1732476. 10.1155/2016/1732476 27774312 PMC5059590

[11] Rawlinson A Elcock C Cheung A Al-Buhairi A Khanna S Walsh TF. An in-Vitro and in-Vivo Methodology Study of Alveolar Bone Measurement Using Extra-Oral Radiographic Alignment Apparatus, Image Pro-Plus Software and a Subtraction Programme. J Dent (2005) 33:781–8. 10.1016/j.jdent.2005.01.013 15922503

[12] Egan KP Brennan TA Pignolo RJ. Bone Histomorphometry Using Free and Commonly Available Software. Histopathology (2012) 61:1168–73. 10.1111/j.1365-2559.2012.04333.x PMC3499639 22882309

[13] Mersmann HJ MacNeil MD. Variables in Estimation of Adipocyte Size and Number With a Particle Counter. J Anim Sci (1986) 62:980–91. 10.2527/jas1986.624980x 3519556

[14] Hassanlou L Meshgini S Alizadeh E. Evaluating Adipocyte Differentiation of Bone Marrow-Derived Mesenchymal Stem Cells by a Deep Learning Method for Automatic Lipid Droplet Counting. Comput Biol Med (2019) 112:103365. 10.1016/j.compbiomed.2019.103365 31374349

[15] Stern MP Conrad F. An Automated, Direct Method for Measuring Adipocyte Cell Size. Clin Chim Acta (1975) 65:29–37. 10.1016/0009-8981(75)90331-9 1192607

[16] Lee JH Kirkham JC McCormack MC Medina MA Nicholls AM Randolph MA. A Novel Approach to Adipocyte Analysis. Plast Reconstr Surg (2012) 129:380–7. 10.1097/PRS.0b013e31823aea29 22286421

[17] Parlee SD Lentz SI Mori H MacDougald OA. Quantifying Size and Number of Adipocytes in Adipose Tissue. Methods Enzymol (2014) 537:93–122. 10.1016/B978-0-12-411619-1.00006-9 24480343 PMC4069255

[18] Osman OS Selway JL Kępczyńska MA Stocker CJ O’Dowd JF Cawthorne MA. A Novel Automated Image Analysis Method for Accurate Adipocyte Quantification. Adipocyte (2013) 2:160–4. 10.4161/adip.24652 PMC3756104 23991362

[19] Zhi X Wang J Lu P Jia J Shen HB Ning G. AdipoCount: A New Software for Automatic Adipocyte Counting. Front Physiol (2018) 9:85. 10.3389/fphys.2018.00085 29515452 PMC5826178

