# Supplementary figures and images for: Combination Usage of AdipoCount and Image-Pro Plus/ImageJ Software for Quantification of Adipocyte Sizes
Source: Front Endocrinol (Lausanne). 2021 Aug 4;12:642000. doi: 10.3389/fendo.2021.642000 (PMC8371441; doi:10.3389/fendo.2021.642000)

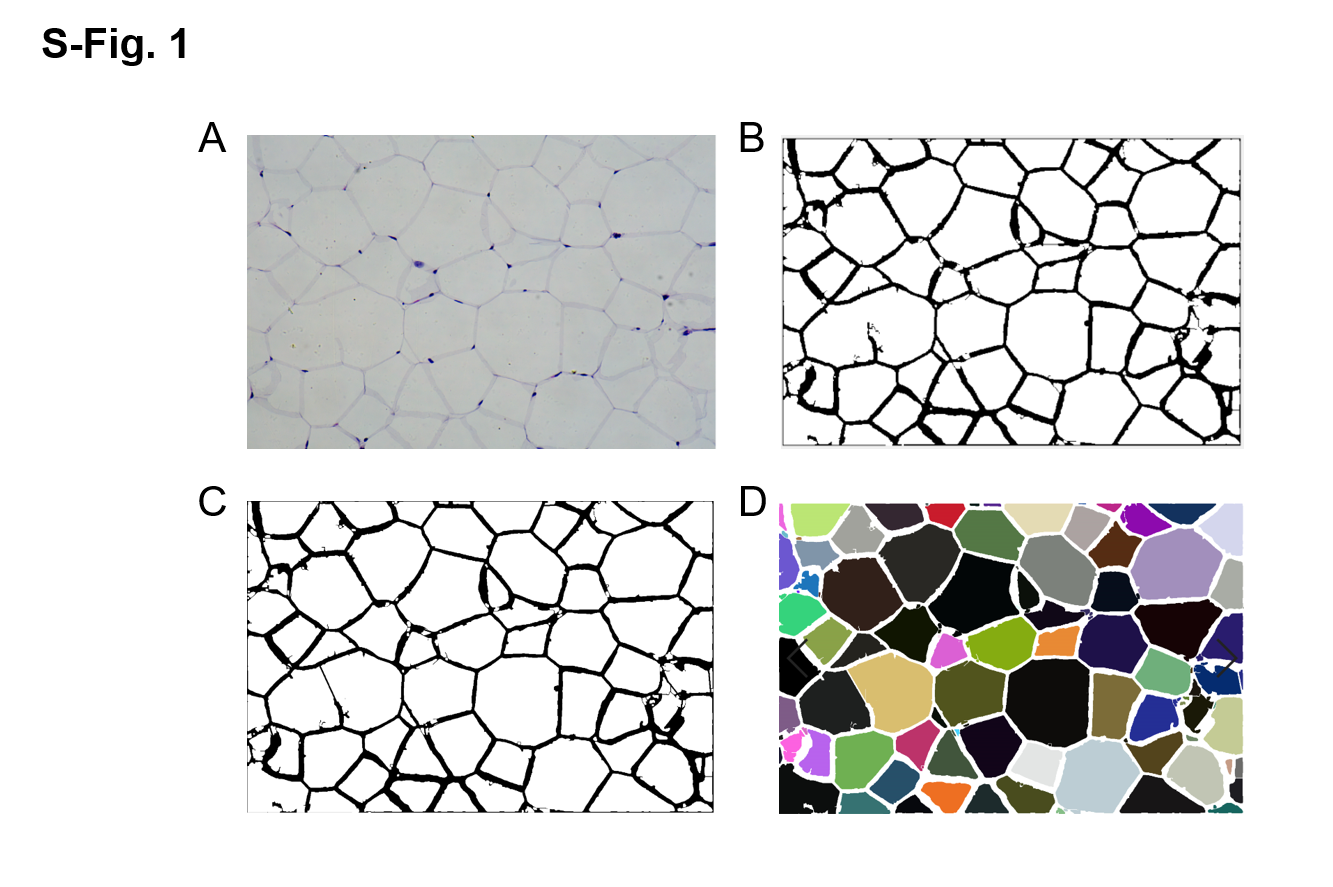

Supplement: Supplementary Figure 1 — Two different segmentation images of AdipoCount. (A) Original image of adipocytes. (B) Preliminary segmentation image without correction. (C) Monochrome segmentation image after correction. (D) Color segmentation image after correction. [file Image_1.tif]

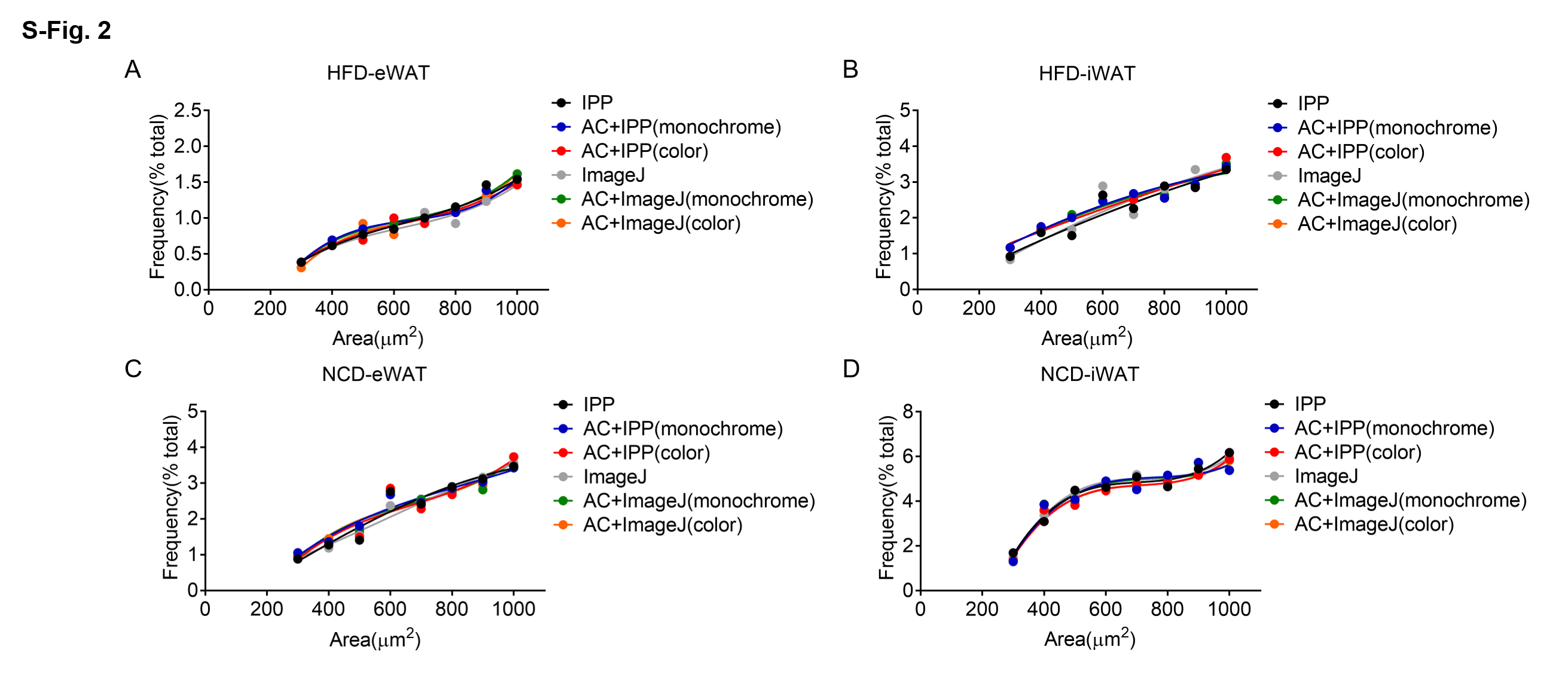

Supplement: Supplementary Figure 2 — The combined methods measured the size of very small adipocyte accurately. (A-D) The size distributions of adipocytes (range of 240-1000µm2, in 100 increments) measured by manual and combined methods, in eWAT of obese mice (A), in iWAT of obese mice (B), in eWAT of lean mice (C), in iWAT of lean mice (D) IPP, Image-Pro Plus; AC, AdipoCount. [file Image_2.tif]

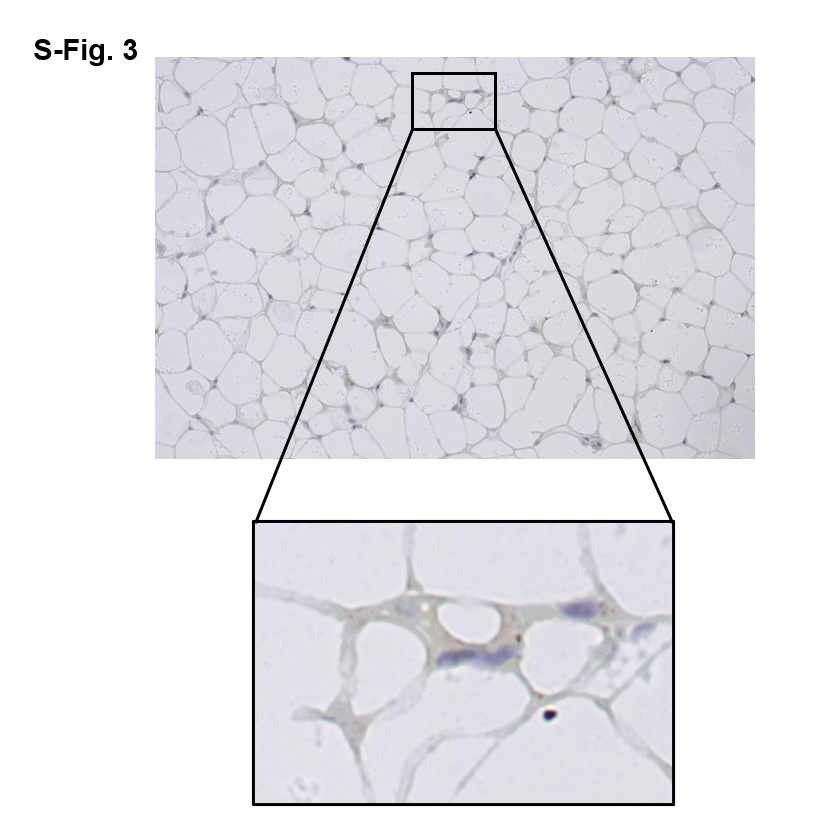

Supplement: Supplementary Figure 3 — UCP-1 immunostaining of iWAT in lean mice. [file Image_3.tif]
