# Supplementary material for: Combination Usage of AdipoCount and Image-Pro Plus/ImageJ Software for Quantification of Adipocyte Sizes
Source: Front Endocrinol (Lausanne). 2021 Aug 4;12:642000. doi: 10.3389/fendo.2021.642000 (PMC8371441; doi:10.3389/fendo.2021.642000)
Supplement: Supplementary file 4 [file Table_1.docx]

**Supplementary Table 1**

**The cell number of each class in eWAT of obese mice**

| HFD-eWAT | Methods | | | | | |
| --- | --- | --- | --- | --- | --- | --- |
| Area (μm^2^)  (μm^2^) | IPP | AC+IPP  monochrome | AC+IPP  color | ImageJ | AC+ImageJ  monochrome | AC+ImageJ  color |
| <500 | 23 | 25 | 23 | 22 | 25 | 24 |
| 500-1000 | 78 | 76 | 76 | 72 | 79 | 75 |
| 1000-1500 | 87 | 84 | 83 | 89 | 86 | 86 |
| 1500-2000 | 101 | 99 | 98 | 100 | 98 | 99 |
| 2000-2500 | 96 | 95 | 95 | 95 | 96 | 86 |
| 2500-3000 | 91 | 90 | 90 | 92 | 90 | 94 |
| 3000-3500 | 84 | 86 | 87 | 84 | 84 | 84 |
| 3500-4000 | 80 | 83 | 78 | 81 | 80 | 78 |
| 4000-4500 | 79 | 82 | 84 | 74 | 76 | 76 |
| 4500-5000 | 75 | 74 | 76 | 78 | 73 | 71 |
| 5000-5500 | 65 | 66 | 67 | 65 | 67 | 69 |
| 5500-6000 | 52 | 54 | 56 | 52 | 54 | 54 |
| 6000-6500 | 48 | 49 | 53 | 48 | 48 | 52 |
| 6500-7000 | 44 | 40 | 43 | 46 | 43 | 47 |
| 7000-7500 | 33 | 36 | 36 | 38 | 41 | 41 |
| 7500-8000 | 43 | 46 | 43 | 42 | 40 | 43 |
| 8000-8500 | 34 | 32 | 35 | 34 | 34 | 34 |
| 8500-9000 | 30 | 34 | 33 | 29 | 31 | 32 |
| 9000-9500 | 27 | 25 | 26 | 30 | 27 | 27 |
| 9500-10000 | 26 | 27 | 27 | 26 | 26 | 27 |
| 10000-10500 | 23 | 24 | 23 | 22 | 22 | 23 |
| 10500-11000 | 17 | 16 | 16 | 17 | 17 | 19 |
| 11000-11500 | 19 | 15 | 13 | 17 | 17 | 15 |
| 11500-12000 | 12 | 11 | 10 | 13 | 13 | 12 |
| 12000-12500 | 8 | 8 | 8 | 9 | 9 | 9 |
| 12500-13000 | 6 | 6 | 5 | 8 | 7 | 7 |
| 13000-13500 | 8 | 7 | 6 | 7 | 7 | 6 |
| 13500-14000 | 5 | 4 | 5 | 4 | 4 | 4 |
| 14000-14500 | 3 | 3 | 3 | 3 | 3 | 3 |
| 14500-15000 | 2 | 2 | 1 | 2 | 2 | 2 |
| Total | 1299 | 1299 | 1299 | 1299 | 1299 | 1299 |
