# Supplementary material for: Combination Usage of AdipoCount and Image-Pro Plus/ImageJ Software for Quantification of Adipocyte Sizes
Source: Front Endocrinol (Lausanne). 2021 Aug 4;12:642000. doi: 10.3389/fendo.2021.642000 (PMC8371441; doi:10.3389/fendo.2021.642000)
Supplement: Supplementary file 5 [file Table_2.docx]

**Supplementary Table 2**

**The cell number of each class in iWAT of obese mice**

| HFD-iWAT | Methods | | | | | |
| --- | --- | --- | --- | --- | --- | --- |
| Area (μm^2^)  (μm^2^) | IPP | AC+IPP  monochrome | AC+IPP  color | ImageJ | AC+ImageJ  monochrome | AC+ImageJ  color |
| <500 | 96 | 118 | 116 | 98 | 118 | 116 |
| 500-1000 | 334 | 336 | 340 | 346 | 336 | 340 |
| 1000-1500 | 367 | 377 | 373 | 363 | 377 | 373 |
| 1500-2000 | 356 | 340 | 330 | 352 | 338 | 330 |
| 2000-2500 | 301 | 281 | 283 | 305 | 281 | 283 |
| 2500-3000 | 227 | 239 | 249 | 229 | 241 | 249 |
| 3000-3500 | 147 | 160 | 141 | 145 | 143 | 141 |
| 3500-4000 | 126 | 116 | 132 | 124 | 132 | 132 |
| 4000-4500 | 120 | 114 | 116 | 120 | 114 | 116 |
| 4500-5000 | 80 | 84 | 84 | 74 | 84 | 84 |
| 5000-5500 | 68 | 54 | 54 | 66 | 54 | 54 |
| 5500-6000 | 42 | 42 | 42 | 42 | 42 | 42 |
| 6000-6500 | 31 | 33 | 33 | 31 | 33 | 33 |
| 6500-7000 | 30 | 32 | 32 | 30 | 32 | 32 |
| 7000-7500 | 18 | 20 | 20 | 22 | 20 | 20 |
| 7500-8000 | 14 | 10 | 10 | 10 | 10 | 10 |
| 8000-8500 | 10 | 12 | 12 | 10 | 12 | 12 |
| 8500-9000 | 13 | 10 | 11 | 11 | 11 | 11 |
| 9000-9500 | 4 | 6 | 6 | 6 | 6 | 6 |
| 9500-10000 | 2 | 3 | 3 | 3 | 3 | 3 |
| 10000-10500 | 2 | 1 | 1 | 1 | 1 | 1 |
| 10500-11000 | 1 | 1 | 1 | 1 | 1 | 1 |
| Total | 2389 | 2389 | 2389 | 2389 | 2389 | 2389 |
