# Supplementary material for: Combination Usage of AdipoCount and Image-Pro Plus/ImageJ Software for Quantification of Adipocyte Sizes
Source: Front Endocrinol (Lausanne). 2021 Aug 4;12:642000. doi: 10.3389/fendo.2021.642000 (PMC8371441; doi:10.3389/fendo.2021.642000)
Supplement: Supplementary file 6 [file Table_3.docx]

**Supplementary Table 3**

**The cell number of each class in eWAT of lean mice**

| NCD-eWAT | Methods | | | | | |
| --- | --- | --- | --- | --- | --- | --- |
| Area (μm^2^)  (μm^2^) | IPP | AC+IPP  monochrome | AC+IPP  color | ImageJ | AC+ImageJ  monochrome | AC+ImageJ  color |
| <500 | 81 | 96 | 87 | 84 | 93 | 91 |
| 500-1000 | 334 | 329 | 333 | 324 | 331 | 333 |
| 1000-1500 | 286 | 297 | 293 | 287 | 295 | 285 |
| 1500-2000 | 290 | 285 | 285 | 291 | 285 | 279 |
| 2000-2500 | 235 | 236 | 229 | 239 | 239 | 236 |
| 2500-3000 | 202 | 188 | 186 | 194 | 180 | 185 |
| 3000-3500 | 167 | 169 | 179 | 177 | 169 | 181 |
| 3500-4000 | 149 | 163 | 159 | 151 | 170 | 159 |
| 4000-4500 | 106 | 99 | 109 | 103 | 101 | 110 |
| 4500-5000 | 95 | 97 | 93 | 103 | 98 | 94 |
| 5000-5500 | 82 | 72 | 76 | 76 | 70 | 78 |
| 5500-6000 | 63 | 61 | 63 | 61 | 61 | 61 |
| 6000-6500 | 61 | 59 | 57 | 59 | 59 | 57 |
| 6500-7000 | 36 | 36 | 38 | 36 | 36 | 38 |
| 7000-7500 | 18 | 22 | 22 | 22 | 22 | 22 |
| 7500-8000 | 21 | 19 | 19 | 17 | 19 | 19 |
| 8000-8500 | 21 | 19 | 19 | 21 | 19 | 19 |
| 8500-9000 | 19 | 15 | 15 | 19 | 15 | 15 |
| 9000-9500 | 3 | 7 | 7 | 7 | 7 | 7 |
| 9500-10000 | 3 | 3 | 3 | 1 | 3 | 3 |
| 10000-10500 | 4 | 4 | 4 | 4 | 4 | 4 |
| 10500-11000 | 2 | 2 | 2 | 2 | 2 | 2 |
| Total | 2278 | 2278 | 2278 | 2278 | 2278 | 2278 |
