# Supplementary material for: Combination Usage of AdipoCount and Image-Pro Plus/ImageJ Software for Quantification of Adipocyte Sizes
Source: Front Endocrinol (Lausanne). 2021 Aug 4;12:642000. doi: 10.3389/fendo.2021.642000 (PMC8371441; doi:10.3389/fendo.2021.642000)
Supplement: Supplementary file 7 [file Table_4.docx]

**Supplementary Table 4**

**The cell number of each class in iWAT of lean mice**

| NCD-iWAT | Methods | | | | | |
| --- | --- | --- | --- | --- | --- | --- |
| Area (μm^2^)  (μm^2^) | IPP | AC+IPP  monochrome | AC+IPP  color | ImageJ | AC+ImageJ  monochrome | AC+ImageJ  color |
| <500 | 357 | 355 | 338 | 354 | 355 | 342 |
| 500-1000 | 1001 | 990 | 968 | 1013 | 985 | 966 |
| 1000-1500 | 881 | 878 | 891 | 880 | 883 | 890 |
| 1500-2000 | 634 | 648 | 645 | 651 | 652 | 651 |
| 2000-2500 | 456 | 438 | 444 | 447 | 439 | 448 |
| 2500-3000 | 265 | 269 | 272 | 263 | 265 | 270 |
| 3000-3500 | 130 | 135 | 139 | 129 | 135 | 136 |
| 3500-4000 | 97 | 89 | 92 | 86 | 95 | 94 |
| 4000-4500 | 47 | 53 | 53 | 50 | 50 | 53 |
| 4500-5000 | 35 | 32 | 42 | 32 | 29 | 38 |
| 5000-5500 | 12 | 15 | 20 | 13 | 15 | 18 |
| 5500-6000 | 9 | 7 | 7 | 6 | 6 | 5 |
| 6000-6500 | 2 | 2 | 2 | 2 | 2 | 2 |
| 6500-7000 | 1 | 1 | 1 | 1 | 1 | 1 |
| Total | 3927 | 3912 | 3914 | 3927 | 3912 | 3914 |
